# Understory Vegetation Regulated the Soil Stoichiometry in Cold-Temperate Larch Forests

**DOI:** 10.3390/plants14071088

**Published:** 2025-04-01

**Authors:** Ruihan Xiao, Xinyuan Liang, Beixing Duan

**Affiliations:** 1 School of Hydraulic and Electric Power, Heilongjiang University, Harbin 150080, China; xiaoruihan@hlju.edu.cn (R.X.); liangxinyuan_lxy@126.com (X.L.); 2 International Joint Laboratory of Hydrology and Hydraulic Engineering in Cold Regions of Heilongjiang Province, Harbin 150080, China

**Keywords:** understory vegetation, soil stoichiometry, Daxing’an Mountains, cold-temperate

## Abstract

Carbon (C), nitrogen (N), and phosphorus (P) are vital nutrients in the soil, exerting a profound influence on the primary productivity of ecosystems. However, our understanding of how the understory influences soil nutrients and their stoichiometry remains limited, especially in cold-temperate forests where the understory plays a crucial role in mediating soil nutrient cycling. To elucidate the effect of understory vegetation on soil nutrients, three typical larch forests, namely S*phagnum*–*Bryum*–*Rhododendron tomentosum*–*Larix gmelinii* forest (SLL), *Rhododendron dauricum*–*Larix gmelinii* forest (RL), and *Rhododendron tomentosum*–*Larix gmelinii* forest (LL), were selected in the typical cold-temperate region of northeast China to determine the soil organic carbon (SOC), total nitrogen (TN), total phosphorus (TP) contents, and their stoichiometric characteristics in 0–100 cm soil depth. The results revealed the following: (1) Significant differences in soil nutrient and its stoichiometry existed among the three different forest types (*p* < 0.001), with the SLL displaying the highest mean SOC, TN, and TP contents, as well as soil C:N, C:P, and N:P ratios, whereas the RL exhibited the lowest values (*p* < 0.05). (2) Across the 0–100 cm soil profile, the soil nutrient content and stoichiometry showed decreasing trends with soil depth, with significant differences among the soil layers. (3) Variations in soil stoichiometry were significantly correlated with soil bulk density, pH, soil temperature, soil water content, total porosity, and capillary porosity (*p* < 0.05). This study underscores the necessity of further consideration of the impact of understory vegetation in future research on soil stoichiometry in forest ecosystems.

## 1. Introduction

Soil, as a fundamental component of ecosystems, plays a crucial role in plant growth and in directly regulating the composition, structure, and productivity levels of vegetation communities. In forest ecosystems, the soil nutrients status and cycle are essential to maintain stability and sustainable development. Soil stoichiometry can reflect the carbon (C), nitrogen (N), and phosphorus (P) cycles, holding significant importance in determining the ecological process responses to global climate change [1,2]. In forest ecosystems, soil C:N:P stoichiometric relationships can indicate the balance of elements and the flow of energy, which are crucial for maintaining soil health, land restoration, and the biogeochemical cycle [3]. The cold-temperate forest ecosystem, as a vital component of global forest ecosystems, plays a pivotal role in mitigating climate change and reducing global biodiversity loss [4]. Therefore, in order to gain a better understanding of the energy and nutrient cycling patterns and explore their essential ecological functions, it is necessary to conduct further research on soil stoichiometry in cold-temperate forest ecosystems.

Currently, numerous studies have investigated forest soil stoichiometric characteristics, and most of them focus on the effects of climate [5,6], altitude [7,8], land utilization [9], and additional factors [10,11,12,13] on soil stoichiometry. Recent research has increasingly emphasized the role of vegetation composition in shaping soil stoichiometry. For example, Hui et al. [14] demonstrated that distinct vegetation types in tropical forests exhibited differential nutrient use efficiencies and environmental adaptability, which directly altered the soil nutrient cycle and its stoichiometric characteristics. Similarity, Feng et al. [5] found that the vegetation on the Qinghai Tibet Plateau indirectly affected the soil nitrogen–phosphorus ratio by regulating soil total nitrogen and phosphorus content. The understory vegetation plays a critical role in regulating the soil nutrient composition [15] and cycling processes in forest ecosystems [16]. Nonetheless, the effect of understory vegetation on soil nutrient distribution and stoichiometry still lacks attention, especially in cold-temperate forests characterized by lower biodiversity and a simplistic structure (typically consisting of a tree layer and an understory of short woody ericaceous shrubs). Thus, exploring the role of understory vegetation in regulating soil stoichiometric attributes in cold-temperate forest ecosystems could deepen insights into the knowledge of the role of understory vegetation in nutrient cycling.

Furthermore, soil depth also has significant effects on soil stoichiometry. Increased soil depth reduces aeration and microbial activity, thereby altering nutrient availability [17]. Simultaneously, there are significant effects on soil microbial activity by plant exudates, with variances in the organic matter content available for microbial decomposition across different soil depths, leading to variations in soil nutrient distribution and stoichiometric traits [18]. Previous studies have explored the effect of vegetation types on the soil stoichiometry in subtropical and temperate regions, unveiling a reduction in soil nutrient content with soil depth, alongside the significant effects of soil depth on soil properties [19,20]. Huang et al. [21] demonstrated close correlations between variations in soil nutrients and stoichiometric ratios in the Xinglong Mountain coniferous-broadleaved mixed forest with vegetation types and soil depth. Plant regulation of soil nutrients exhibits depth-dependent mechanisms, as follows: in surface layers, fine root turnover dominates nutrient cycling through rapid carbon inputs and mycorrhizal associations [22], whereas deeper soils primarily rely on the slow decomposition of woody roots and ligand-assisted leaching processes mediated by deep-rooted vegetation [23]. Thus, it is essential to explore the interaction effects of soil depth and plant types on soil stoichiometry. Nevertheless, these studies primarily focus on tropical, subtropical, and temperate regions, with limited studies on the stoichiometry across different soil depths in cold-temperate forest ecosystems. In addition, most of the existing studies have only pointed out that the stoichiometric characteristics of the surface soil were related to understory vegetation and environmental factors. There are few studies on the stoichiometric characteristics in deep soil. Consequently, exploring the influence of understory vegetation on soil nutrients across depths can provide a comprehensive analysis of understory vegetation impact on the stoichiometric characteristics of forest ecosystem soils.

The northern part of the Daxing’an Mountains is a key area in the China Natural Forest Protection Program and the only high-latitude cold-temperate forests with vast continuous permafrost [24,25,26]. The forest ecosystems are mainly dominated by *Larix gmelinii* forests. However, considerable diversity exists in the composition of understory vegetation. Studies have suggested that variations in understory vegetation types could affect the soil physicochemical properties in larch forests [27]. However, the specific influence of understory vegetation on soil stoichiometry and its primary influencing factors remains unclear. Therefore, in this study, three different types of larch forest in the northern Daxingʹan Mountains, composed of the same tree layer but different understory vegetation, were selected, namely *Rhododendron dauricum* –*Larix gmelinii* forest (RL), *Rhododendron tomentosum*–*Larix gmelinii* forest (LL), and S*phagnum*–*Bryum*–*Rhododendron tomentosum*–*Larix gmelinii* forest (SLL). Soil organic carbon (SOC), total nitrogen (TN), total phosphorus (TP), and their stoichiometric characteristics across three different types of larch forest were analyzed to achieve the following: (1) explore the effect of understory vegetation composition on soil nutrient cycling; and (2) identify the key factors regulating soil stoichiometric relationships. We hypothesized the following: (1) the understory vegetation will significantly affect soil nutrients and their stoichiometric characteristics; and (2) these effects differ across soil layers and are more pronounced in the surface soil layer.

## 2. Results

### 2.1. Soil Properties Among Different Forest Types

**Figure 1:**
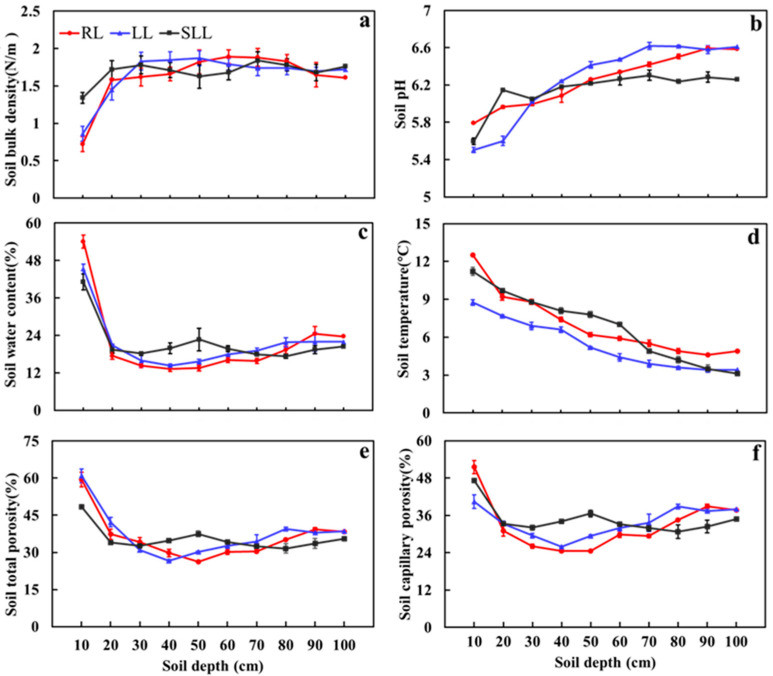
Soil bulk density (**a**), pH (**b**), water content (**c**), temperature (**d**), total porosity (**e**), and capillary porosity (**f**) in the three types of larch forest ecosystems. Note: LL: *Rhododendron tomentosum*–*Larix gmelinii* forest; RL: *Rhododendron dauricum*–*Larix gmelinii* forest; and SLL: S*phagnum*–*Bryum*–*Rhododendron tomentosum*–*Larix gmelinii* forest. Data are means ± standard deviation (*n* = 5).

As shown in Figure 1, the soil bulk density, pH value, temperature, water content, total porosity, and capillary porosity in the larch forest ranged from 0.72 to 1.89 g·cm^−3^, 5.50 to 6.62, 3.10 to 12.50 °C, 13.27% to 54.08%, 26.29% to 60.97%, and 24.53% to 51.53%, respectively. The soil bulk density and pH value showed a significant increase with soil depth. In contrast, the soil temperature, water content, total porosity, and capillary porosity also showed a significant increasing trend with soil depth (*p* < 0.05). The forest type significantly affected soil bulk density, pH value, and capillary porosity (*p* < 0.05).

### 2.2. Soil Nutrient Content Among Different Forest Types

**Figure 2:**
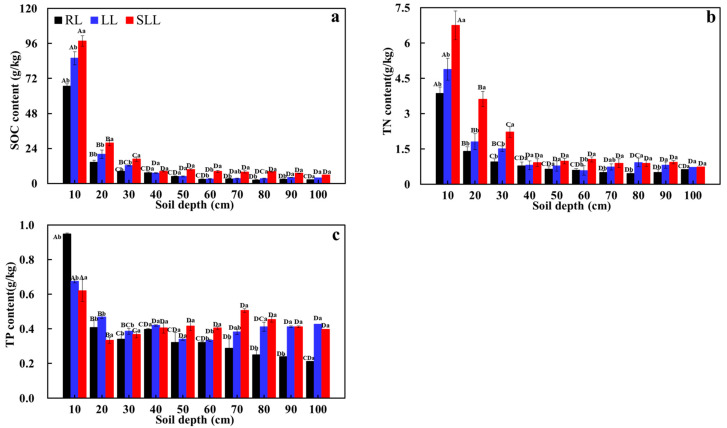
Soil organic carbon (**a**), total nitrogen (**b**), and total phosphorus (**c**) contents in the three types of larch forest ecosystems. Note: LL: *Rhododendron tomentosum*–*Larix gmelinii* forest; RL: *Rhododendron dauricum*–*Larix gmelinii* forest; and SLL: S*phagnum*–*Bryum*–*Rhododendron tomentosum*–*Larix gmelinii* forest. Different capital letters represent significant differences among different layers in the soil and different lowercase letters represent significant differences among forest types (*p* < 0.05). Data are means ± standard deviation (*n* = 5). SOC: soil organic carbon; TN: soil total nitrogen; TP: soil total phosphorus.

The range of variations in SOC, TN, and TP contents in the larch forest were 2.39–97.71 g·kg^−1^, 0.46–6.76 g·kg^−1^, and 0.21–0.95 g·kg^−1^, respectively. The understory vegetation, soil depth, and their interaction all had significant effects on the SOC, TN, and TP contents (*p* < 0.001, Table 1). The SOC, TN, and TP contents in the RL, LL, and SLL all showed a sharp decrease from 0 to 30 cm soil depth, followed by a fluctuating decline from 30 to 100 cm soil depth. The average SOC, TN, and TP contents in the 0–100 cm soil layer among the three types of larch forests were RL < LL < SLL (*p* < 0.05) (Figure 2).

### 2.3. Soil Stoichiometric Ratios of Different Forest Types

**Figure 3:**
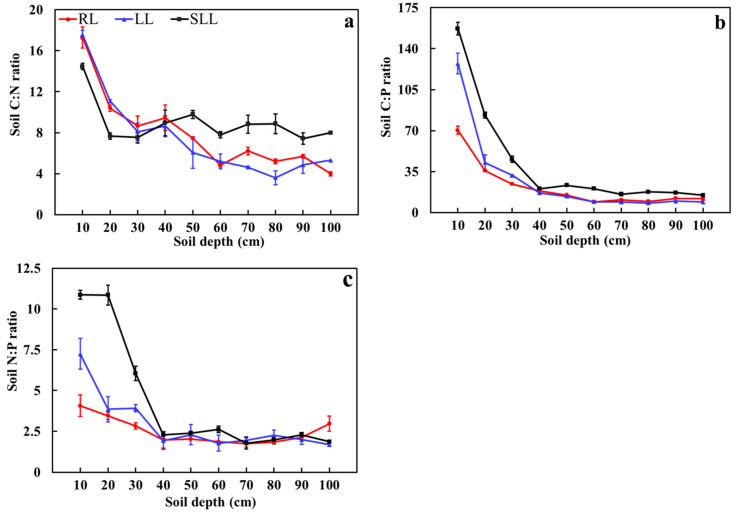
Stoichiometric characteristics of soil C:N (**a**), C:P (**b**), and N:P (**c**) in the three types of larch forest ecosystems. Note: LL: *Rhododendron tomentosum*–*Larix gmelinii* forest; RL: *Rhododendron dauricum*–*Larix gmelinii* forest; and SLL: S*phagnum*–*Bryum*–*Rhododendron tomentosum*–*Larix gmelinii* forest. Data are means ± standard deviation (*n* = 5).

The range of soil C:N, C:P, and N:P ratios in the larch forest were 3.60–17.56, 8.12–157.17, and 1.68–10.87, respectively. The understory vegetation, soil depth, and their interactions had significant effects on the soil stoichiometric ratios (*p* < 0.01, Table 2). In the RL, LL, and SLL forests, the soil C:N, C:P, and N:P ratios sharply decreased from 0 to 40 cm soil depth and then gradually stabilized at lower levels from 40 to 100 cm soil depth. The average soil C:N, C:P, and N:P ratios in the 0–100 cm soil layer among the three types of larch forests were RL < LL < SLL (*p* < 0.05, Figure 3).

### 2.4. Effect of Environmental Factors on Soil Stoichiometric Ratios

**Figure 4:**
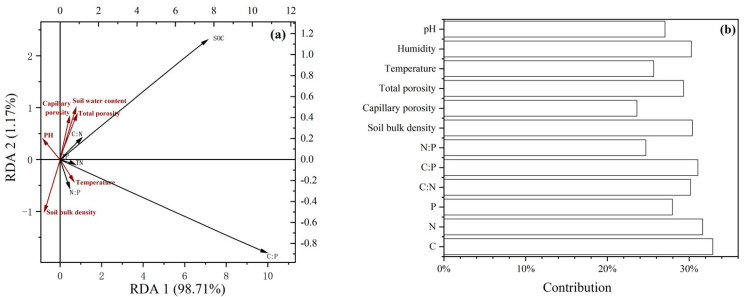
Results of redundancy analysis (**a**) and contribution rate of each factor (**b**). Note: SOC: soil organic carbon; TN: soil total nitrogen; TP: soil total phosphorus.

**Figure 5:**
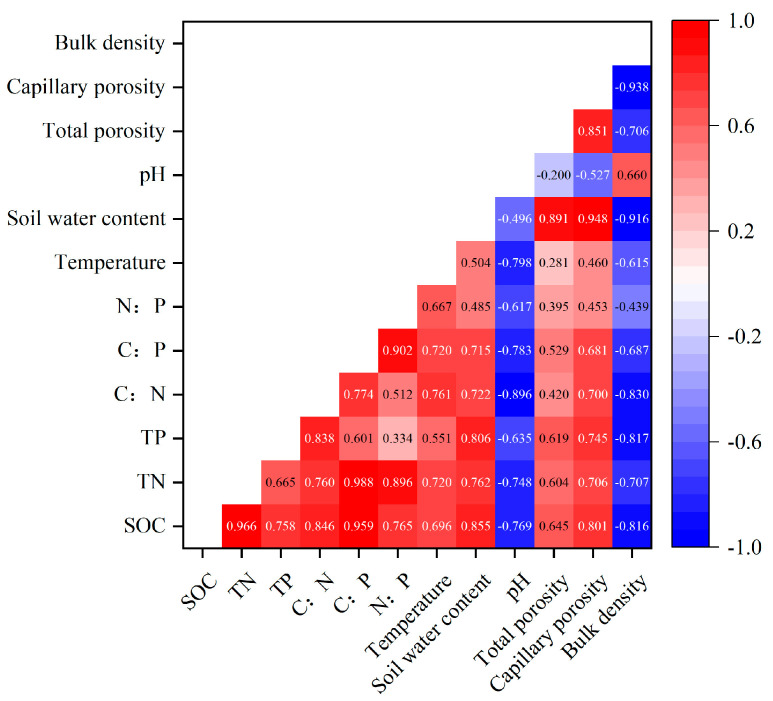
Correlation between SOC, TN, TP contents, and stoichiometric ratios with soil physical properties. Note: SOC: soil organic carbon; TN: soil total nitrogen; TP: soil total phosphorus.

According to the redundancy analysis (RDA) results between the soil stoichiometric characteristics and environmental factors (Figure 4), the cumulative contribution of variances of the environmental factors to soil stoichiometric variations was 99.88% for the first two axes, and RDA1 and RDA2 explained the variations of 98.71% and 1.17%, respectively. Soil bulk density and water content had largest effects on soil nutrient content and stoichiometric characteristics compared to the other environmental factors (Table A1). According to Figure 5, the soil stoichiometric characteristics were significantly negatively correlated with soil bulk density and pH, and significantly positively correlated with temperature, water content, total porosity, and capillary porosity (*p* < 0.05).

## 3. Discussion

### 3.1. Influence of Understory Vegetation on Soil Nutrient Content

Understory vegetation plays a pivotal role in mediating forest ecosystem processes, including successional dynamics, soil nutrient cycling, and biodiversity conservation [28]. In this study, the average SOC, TN, and TP contents in the 0–100 cm soil layer all ranged SLL > LL > RL (Figure 2) (*p* < 0.05), consistent with our first hypothesis. This result emphasizes the important role of understory vegetation in regulating soil nutrients. This may be due to the unique traits of the different understories, which affect the litter quality, quantity, and decomposition rate, thus influencing soil nutrient content and distribution [29]. Additionally, different understory vegetation types exhibit different rhizosphere effects, which can affect geochemical and biological processes, and ultimately lead to differences in soil nutrient distribution [15]. Notably, although *Rhododendron dauricum*–*Larix gmelinii* and *Rhododendron tomentosum*–*Larix gmelinii* are both shrubs of the *Ericaceae* family, the soil nutrient content was still different. This may be because the leaves of *Rhododendron dauricum* are leathery and difficult to decompose, which hinders the input of organic matter decomposition products into the soil, resulting in lower soil nutrient content [30].

There were significant differences in the SOC, TN, and TP contents among the different soil layers in the three types of larch forest (Figure 2). The soil nutrient content in the RL, LL, and SLL was highest in the surface soil layer (0–30 cm) and then decreased sharply. This phenomenon may be mainly due to the following two reasons: Firstly, with increasing soil depth, the distribution of plant roots decreases, reducing nutrient sources and causing differences in soil nutrient content [31]. Secondly, litter, as an important source of soil nutrients, gradually returns the nutrients to the soil through microbial decomposition, contributing to soil nutrient accumulation and turnover, resulting in a higher nutrient content in the surface layer than that found in the deeper layers. Furthermore, with increasing soil depth, the variations in SOC and TN contents were significantly higher than those of the soil TP content, indicating more obvious surface aggregation. This result may be due to the fact that, in addition to the litter decomposition, the soil TP content is also influenced by rock weathering. However, rock weathering is a slow and stable process, which can result in relatively small variations in soil TP content [32].

### 3.2. Influence of Understory Vegetation on Soil Stoichiometry

In this study, the soil stoichiometric ratios also showed significant differences among the different forest types, consistent with the findings of Xiao et al. [33]. The average C:N ratio of terrestrial soils in China was 11.9 [34], and the average N:P ratio was 5.2 [35]. The surface soil C:N ratio in the SLL was similar to the average level of terrestrial soils in China, but the N:P ratio was significantly higher. This indicates low phosphorus availability and high nitrogen availability in the surface layer soil in the SLL. The C:N ratio is inversely proportional to the amount of available nitrogen released during the mineralization of organic matter, meaning that the larger the C:N ratio, the less available nitrogen is released [36]. Therefore, the decomposition of organic matter and the potential for available nitrogen release were relatively high in the surface soil in the SLL. Previous studies have shown that soil N:P can be used to determine the type of nutrient limitation [37]. When the N:P < 10, it is mainly limited by nitrogen; when N:P > 20, it is mainly limited by phosphorus; and when 10 < N:P < 20, it is limited by both nitrogen and phosphorus [37]. The N:P ratios in the surface layer of the LL and RL were both below 10, whereas, in the SLL, it was slightly above 10. This suggests that the surface soil in the SLL was relatively less limited by nitrogen, while the LL and RL were more strongly limited by nitrogen. Soil N:P is widely used in ecology as a diagnostic indicator to determine the threshold of nutrient limitation [38]. Generally, soil C:P can reflect the availability of phosphorus in the soil [39]. The C:P ratio in the SLL was significantly higher than that observed in the other two forest types, indicating higher phosphorus availability in the SLL. Our study also found that there were significant differences in soil C:P and N:P among the three forest types. This may be due to the different absorption and release of major elements from the atmosphere and soil by different plants, leading to variations in soil C:P and N:P under the different vegetation types [40].

Soil depth also had significant effects on soil C:N in our study, as it decreased with increasing soil depth, consistent with the findings of Ji et al. [41] and Chen et al. [42]. Soil C:N was mainly influenced by the quality and quantity of litter input. Higher litter input to the soil can provide a sufficient substrate for microbial decomposition, thus inducing higher nutrient availability [43]. With increasing soil depth, soil C:P showed a significantly decreasing trend, indicating that soil depth significantly affects soil stoichiometric characteristics. Additionally, consistent with our second hypothesis, soil N:P showed a sharp decline trend in the 0–30 cm soil layers, followed by stabilization with increasing soil depth, which was contrary to the findings of Chen et al. [42] on the soil N:P of the *Larix principis*–*rupprechtii* forests of different ages. This difference may be related to the soil, vegetation, and climatic factors in different regions. The plant in Chen’s study was limited by phosphorus, while our study area was severely nitrogen-limited. The decrease in soil N:P with the increase in soil layer indicates that the soil nitrogen limit is more serious. This suggests that the role of nitrogen limitation became stronger as the soil layer increased. Song et al. [44] pointed out that the world soil N:P level is 13.1. The soil N:P in our study was lower than 13.1, further supporting the previous discussion on nitrogen limitation. Elisabeth et al. [45] pointed out that soil was N-limited in Australia when soil N:P < 10. From the larger theoretical frameworks, it is of great significance to study the influence of soil stoichiometric characteristics on clarifying the nutrient balance, plant nutrient limitation mechanism, and chemical cycling process in forest soil ecosystems [46].

### 3.3. Relationship Between Soil Stoichiometric Characteristics and Environmental Factors

In this study, SOC content, TN content, and C:P and N:P ratios showed significant negative correlations with soil bulk density and pH, consistent with previous studies [47]. The redundancy analysis results also indicated that soil bulk density and soil water content had stronger effects on soil nutrient content and stoichiometric characteristics than the other environmental factors. This may be due to the higher soil bulk density leading to lower soil porosity, which can inhibit the circulation of soil water content and gases, as well as the activity of plant roots, thereby negatively affecting the soil carbon and nitrogen content. The soil water content directly influences the growth and activity of soil microorganisms and the absorption of soil nutrients by plant roots, thus impacting the soil stoichiometric characteristics. In this study, the soil pH showed a significant correlation with the soil nutrient stoichiometric ratios, contrary to the conclusions of Wang et al. [48] regarding the effect of soil pH on carbon, nitrogen, and phosphorus ecological stoichiometry. Additionally, Zhang et al. [49] pointed out that there were no significant correlations between soil pH and the stoichiometric characteristics of carbon, nitrogen, and phosphorus in the soil of *Ziziphus jujuba* Mill. orchards in the Tarim Basin. This suggests that the impact of soil pH on soil nutrient stoichiometric characteristics varies greatly under different environmental conditions, highlighting the necessity of analyzing the relationship between soil pH and stoichiometric characteristics for different understory vegetation types. Soil is the material basis for maintaining the growth and development of trees in forest ecosystems, and its physical and chemical properties and fertility affect and control the health of trees [50]. Understanding the physical and chemical properties of forest soil is important to promote forest ecological benefits [51].

### 3.4. Insights

The hypothesis presented in the introduction has been confirmed. However, several limitations remain. The interaction between plants, soil, and the environment is complex and long term. All of the samples in our study were collected only once, which may overlook the dynamic fluctuations of forest soil nutrients over time. Although changes in soil stoichiometry are often attributed to variations in understory vegetation, the potential effect of an alteration in soil properties on vegetation structure should not be overlooked. Given that the interaction between plants and soil is a long-term and complex process, long-term field work is needed to better reveal the relationship between the understory in regulating soil nutrients and its stoichiometric characteristics.

Our study is also important for forest management. In recent years, due to the strong impact of human activities, the speed and scale of nitrogen and phosphorus cycling have suffered unprecedented changes [52]. Our study highlights the presence of nutrient limitations within cold-temperate forest systems. This insight aids in more accurately identifying the issues within the carbon, nitrogen, and phosphorus cycles of these forests, thereby contributing to a deeper understanding of their ecological dynamics and potential areas for intervention.

## 4. Materials and Methods

### 4.1. Study Area Description

**Figure 6:**
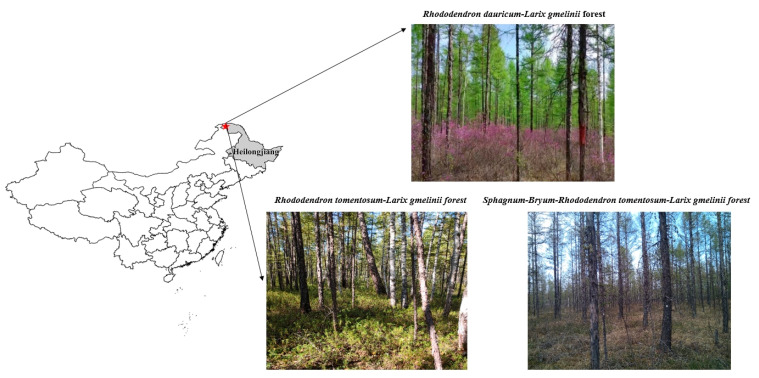
Representation of the study site.

The research site was located in the Heilongjiang Mohe Forest Ecosystem National Positioning Observation and Research Station (122°07′–122°27′ E, 53°22′–53°30′ N) (Figure 6), in the northern Daxingʹan Mountains in northeastern China, bordering Russia. The average annual temperature is −4.9 °C and average annual precipitation ranges from 350 to 500 mm, characterized by a classic cold-temperate continental monsoon climate [26]. The frost-free period lasts for 80–90 days. The predominant soils are classified as brown coniferous forest soils, with additional variations, including marsh soils, meadow soils, and peat soils. The proportion of sand particles in the soil is relatively high, often with gravel. The soil layer is usually shallow (30–40 cm). The biomass of the litter layer ranges from 5.27 to 6.97 Mg·ha^−1^ [53].

The forest coverage in the study area was 75–82%, primarily consisting of cold-temperate bright coniferous forests dominated by *Larix gmelinii*, with associated species such as *Populus davidiana*, *Pinus sylvestris var. mongolica*, and *Betula platyphylla*. The understory shrubs were *Rhododendron dauricum*, *Rhododendron tomentosum*, and *Vaccinium vitis-idaea* [54], respectively.

### 4.2. Experimental Design

The experiment was carried out according to the two-factor completely randomized design [55]. The two factors were the understory vegetation type and the soil depth. Three typical larch forest ecosystems were selected—*Sphagnum*–*Bryum*–*Rhododendron tomentosum*–*Larix gmelinii* forest (SLL), *Rhododendron dauricum*–*Larix gmelinii* forest (RL), and *Rhododendron tomentosum*–*Larix gmelinii* forest (LL)—in July 2020 in the Daxing’an Mountains. In each forest ecosystem, five 20 m × 20 m plots were randomly established to measure the tree height and diameter at the breast height of the trees. The stand type, age, density, elevation, slope, and understory species composition of the selected plots in the larch forest ecosystems were recorded (Table 3).

### 4.3. Soil Sampling

In each plot, five soil sampling points were evenly arranged in an “S” shape, with a straight-line distance of 5 m between adjacent points and 2 m from the tree trunks. After removing the surface litter, a 100 cm soil profile was excavated at each sampling point, and soil samples were collected at ten depths (0–10 cm, 10–20 cm, 20–30 cm, 30–40 cm, 40–50 cm, 50–60 cm, 60–70 cm, 70–80 cm, 80–90 cm, and 90–100 cm). Samples from the same depth in the same forest type plot were mixed in equal proportions and air-dried at room temperature (25 °C). The mixture was crushed and passed through a 2 mm sieve to remove litter, fine roots, and gravel. Additionally, soil samples were collected at each point for the ten depths to determine the soil bulk density and water content.

### 4.4. Soil Analysis

The soil total nitrogen (TN) content was determined using the semi-micro Kjeldahl method, the soil organic carbon (SOC) content was determined using the dichromate oxidation method, and the soil total phosphorus (TP) content was determined using the perchloric-acid–sulfuric-acid method with an ultraviolet spectrophotometer (UV-2550, Kyoto, Japan) [56,57]. The soil water content (%) was measured gravimetrically by oven-drying the whole soil sample at 105 °C for 24 h [58]. The soil pH was determined at a soil-to-water mass ratio of 1:2.5 by using a pH meter (PHS–3C, Shanghai, China) equipped with a calibrated combined-glass electrode [59]. The soil porosity and soil bulk density were determined using the cutting ring method, with an inner diameter of 5.0 cm and a height of 5.0 cm [60].

### 4.5. Statistical Analysis

One-way ANOVA was used to analyze the differences in SOC, TN, and TP contents and stoichiometric characteristics across the different forest types. The post hoc tests with Duncan multiple range tests were conducted for multiple comparisons. Two-way ANOVA was used to study how the interactions between the different factors affect other soil stoichiometric characteristics. The correlations between the SOC, TN, and TP contents, stoichiometric characteristics, and environmental factors were determined using the Pearson test and redundancy analysis. The data processing and analysis in this study were conducted using Microsoft Excel 2022, SPSS 26.0 for Windows, and Origin 2021, with statistical significance set at *p* < 0.05 or *p* < 0.01.

## 5. Conclusions

Our study conducted a comparative analysis of the soil nutrient stoichiometric characteristics from 0 to 100 cm depth in three different types of larch forests. It was found that the variations in forest soil stoichiometric characteristics were closely related to the understory vegetation types and soil depths. Additionally, the soil physical and chemical properties significantly affected its stoichiometric characteristics, and the soil bulk density and water content were more important factors in regulating the nutrient content and stoichiometric ratios than the other factors. In summary, there were significant differences in the stoichiometric characteristics of soils in cold-temperate larch forests with different understory vegetation types. Focusing more on the impact of understory vegetation types on soil stoichiometry can help us to better understand the role of vegetation in improving soil quality.

## Figures and Tables

**Table 1:** Analysis of variance between understory vegetation types and soil depth on soil nutrient content. SOC: soil organic carbon; TN: soil total nitrogen; TP: soil total phosphorus.

Source of Difference	SOC				TN				TP			
	df	F	*p*	Mean Squares	df	F	*p*	Mean Squares	df	F	*p*	Mean Squares
Understory vegetation type	1	85.849	<0.001	995.849	1	97.916	<0.001	11.327	1	24.619	<0.001	0.052
Soil depth	9	29.052	<0.001	337.007	9	4.325	<0.001	0.500	9	46.212	<0.001	0.098
Soil depth × Understory vegetation type	9	9.679	<0.001	112.272	9	11.853	<0.001	1.371	9	19.498	<0.001	0.041

**Table 2:** Analysis of variance between understory vegetation types and soil depth on soil stoichiometric ratios.

Source of Difference	Soil C:N				Soil C:P				Soil N:P			
	df	F	*p*	Mean Squares	df	F	*p*	Mean Squares	df	F	*p*	Mean Squares
Understory vegetation type	1	9.019	0.004	14.786	1	159.614	<0.001	5861.774	1	88.527	<0.001	49.232
Soil depth	9	24.792	<0.001	40.645	9	2.590	0.012	95.131	9	3.344	0.002	1.859
Soil depth × Understory vegetation type	9	6.417	<0.001	10.521	9	30.057	<0.001	1103.821	9	24.324	<0.001	13.527

**Table 3:** Forest ecological characteristics.

Forest Ecological Characteristics	Mean DBH (cm)	Stand Age	Mean Tree Height (m)	Slope/°	Stand Density (Trees·ha^−1^)	Elevation (m)	Understory Species Composition	Coverage (%)
RL	13.78 ± 2.12	75–90	17.23 ± 1.54	3	1266 ± 126	324	*Vaccinium uliginosum* L.	30
							*Ledum palustre* L.	20
							*Vaccinium vitis-idaea* L.	10
							*Rhododendron dauricum* L.	80
LL	13.14 ± 2.61	75–90	16.78 ± 1.94	4	1300 ± 100	326	*Pyrola incarnate H.Andr.*	40
							*Vaccinium uliginosum* L.	20
							*Rhododendron tomentosum* L.	70
							*Vaccinium vitis-idaea* L.	50
							*Fragaria orientalis Losinsk*	10
SLL	14.06 ± 2.23	75–90	17.47 ± 1.60	2	1117 ± 126	332	*Fragaria orientalis Losinsk*	5
							*Vaccinium vitis-idaea* L.	30
							*Pyrola incarnata H.Andr*	10
							*Vaccinium uliginosum* L.	50
							*Vaccinium macrocarpon* L.	20
							*Rhododendron tomentosum* L.	70
							*Bryum* L.	40
							*Sphagnum palustre* L.	60

## Data Availability

The data that support the findings of this study are available from the corresponding author upon reasonable request.

## Appendix A

**Table A1:** The first two axes on the RDA.

**Factors**	**RDA1**	**RDA2**
Soil bulk density	−0.78243	−0.46915
Capillary porosity	0.60729	0.41123
Total porosity	0.77187	0.41938
Temperature	0.76469	−0.23741
Water content	0.81537	0.49641
pH	−0.83584	0.19071
C	8.16872	1.23558
N	0.47731	−0.00935
P	0.03728	0.01977
C:N	1.06701	0.17928
C:P	11.52941	−0.87821
N:P	0.62899	−0.24703
